# War's Effect on Relative Cost of London Hospitals

**Published:** 1917-01-13

**Authors:** 


### KING EDWARD'S HOSPITAL FUND STATISTICS.

### War's Effect on Relative Cost of London Hospitals.

THE report of the Distribution Committee of the King's Fund reveals the fact that in the allocation of grants the relative positions of hospitals in their groups is duly noted by those responsible for the apportionment of the large sum within the gift of the Fund. In respect of one hospital the Committee " regret to see that the cost has increased in greater proportion than that at similar hospitals." From the wording of this note it is to be surmised that the cost per out-patient attendance as well as the cost per occupied bed is taken into consideration. This is as it should be, for under our present system of accountancy, there being no one general accountant for the London hospitals or for any group of hospitals, it is left to the judgment of each institution how much of the annual expenses are debited to in-patients and out-patients respectively. Hence we find the very curious result that within a group a hospital with the lowest average cost per occupied bed has the highest cost per out-patient attendance. The following table illustrates this point amongst others:

RELATIVE POSITIONS AS TO AVERAGE ANNUAL COST PER OCCUPIED BED AND PER OUT-PATIENT ATTENDANCE OF A GROUP OF HOSPITALS UNDER WAR CONDITIONS AS COMPARED WITH THE MOST RECENT NORMAL YEAR.

| | Cost per Occupied Bed | | | Cost per Out-patient Attendance | | |
|---|---|---|---|---|---|---|
| | War Conditions 1915 | Normal Working, 1913 | Increase or Decrease | War Conditions 1915 | Normal Working, 1913 | Increase or Decrease |
| | £ s. d. | £ s. d. | £ s. d. | d. | d. | d. |
| St. George's ... ... | (1) 110 16 7 | (1) 96 6 4 | +14 10 3 | (4) 9·63 | (5) 8·93 | +0·7 |
| London ... ... | (2) 107 17 10 | (2) 93 6 1 | +14 11 9 | (2) 11·61 | (3) 9·50 | +2·11 |
| Westminster ... ... | (3) 102 16 9 | (4) 89 17 9 | +12 19 0 | (3) 10 | (4) 9·47 | +0·53 |
| St. Mary's ... ... | (4) 99 7 0 | (7) 84 10 1 | +14 16 11 | (1) 12·32 | (2) 10·20 | +2·12 |
| Royal Free ... ... | (5) 99 6 4 | (3) 92 10 11 | + 6 15 5 | (5) 9·45 | (6) 8·92 | +0·53 |
| Guy's ... ... ... | (6) 97 19 9 | (6) 87 17 1 | +10 2 8 | (9) 5·86 | (9) 6·19 | −0·33 |
| Middlesex ... ... | (7) 95 14 1 | (8) 78 4 11 | +17 9 2 | (8) 6·41 | (8) 7·15 | −0·74 |
| Charing Cross ... ... | (8) 90 1 0 | (5) 89 11 8 | + 0 9 4 | (6) 9·13 | (7) 8·81 | +0·32 |
| University College ... | (9) 68 15 6 | (9) 71 0 2 | − 2 4 8 | (7) 8·84 | (1) 10·29 | −1·45 |

NOTE.—The figures in brackets indicate relative positions for the year.

In preparing this table we have taken, for comparison with the figures of 1915, a year when the country was at peace for the whole twelve months. The result is curious; with considerable increases in the price of all articles common to the use of hospitals and ordinary households, and an extraordinary increase in the cost of some articles more or less peculiar to the use of hospitals, such as some drugs, indiarubber sheeting, etc., dressings, and glassware, together with higher rates for labour of all kinds, one would confidently expect that any hospital working at full pressure must infallibly show a higher cost per occupied bed, and probably a higher cost per out-patient attendance. But in the field of economics so many things predicted to result through the war have not come to pass, and the general effect of the times upon hospitals is as variable as it is upon other things. We find in the group six hospitals where there has been a substantial increase in the cost per occupied bed, although two of them show a decrease in the out-patient cost, one other with quite a moderate increase under both headings, another practically stationary, and one where is to be found actually a decrease in cost under both in-patients and out-patients.

### THE FIGURES OF UNIVERSITY COLLEGE HOSPITAL.

The phenomenal result of the statistical results as applied to University College requires closer examination. This is the institution presenting the curious financial result referred to in our first paragraph. One point helping in an explanation is that there was an average of forty-three more beds occupied in 1915 than in 1913, and there were 169,645 out-patient attendances, as against 105,217. Naturally, the large standing expenses of the hospital being divided amongst a much larger number of units have a marked effect upon the average cost each represents. But a large proportion of these standing charges under the heading of "Establishment," such as insurance, renewals and repairs of building, and annual cleaning, are not included in the King's Fund calculations. However, other charges which are included —for instance, many of the salaries of the higher officers and management expenses— would not necessarily increase with the number of patients and, being divided by a larger dividend, would naturally tend to reduce the average. Looking at the separate headings of expenditure at University College Hospital, we find that in respect of the average cost per bed under the heading of " Provisions " there is a 10 per cent. increase; under " Surgery and Dispensary " there is a decrease of about 20 per cent., and here the question of stocks is an unknown factor. On a rising market it is possible that the chief pharmacist prudently stocked his dispensary during 1914 to an extent that would not be thought of in normal times. This is one of the weak points of hospital accounts, as governed by the King's Fund, and one that we have on several occasions called attention to; it is a factor that within a given year may easily make a difference of several pounds per bed in a hospital of the size of University College. To continue, under "Domestic" there is the rise we could have expected where the heating, lighting, and cleaning of a large institution are concerned, and the decrease of 10 per cent. under "Salaries and Wages" may illustrate what is a fact generally admitted by those who are in a position to know, that the individual hospital worker is doing far more work during the present shortage of efficient labour than the public generally appreciate. The fact that a penny more or less per out-patient attendance makes a difference of over £2 on the occupied bed at University College Hospital strengthens the argument for a "flat rate" for out-patients, and the appointment of a general auditor or accountant.

Many of the remarks applying to University College apply equally to Charing Cross. The bed occupancy has risen from 141 to 205, while the number of out-patient attendances has remained stationary. Here, again, provisions have risen, and "Surgery and Dispensary" and "Salaries and Wages" have fallen, but under "Domestic" there has been an increase of £1 16s. 6d. per bed.

### A Statistical Deadlock.

But in all these speculations on hospital statistics we come from time to time to the obstacle of the unknown quantity. How can we say whether the cost under "Provisions" is high or low when we are not told how many mouths there are to feed, and how can we judge the expenditure on drugs and dressings when no data is afforded as to stocks, and, consequently, for what period the outlay on material has been made?

